# Clinical importance of the EMSY gene expression and polymorphisms in ovarian cancer

**DOI:** 10.18632/oncotarget.24878

**Published:** 2018-04-03

**Authors:** Agnieszka Dansonka-Mieszkowska, Lukasz M. Szafron, Joanna Moes-Sosnowska, Mariusz Kulinczak, Anna Balcerak, Bozena Konopka, Magdalena Kulesza, Agnieszka Budzilowska, Martyna Lukasik, Urszula Piekarska, Iwona K. Rzepecka, Joanna Parada, Renata Zub, Barbara Pienkowska-Grela, Radoslaw Madry, Jan K. Siwicki, Jolanta Kupryjanczyk

**Affiliations:** ^1^ Department of Pathology and Laboratory Diagnostics, Maria Sklodowska-Curie Institute - Oncology Center, Warsaw, Poland; ^2^ Department of Immunology, Maria Sklodowska-Curie Institute - Oncology Center, Warsaw, Poland; ^3^ Department of Molecular and Translational Oncology, Maria Sklodowska-Curie Institute - Oncology Center, Warsaw, Poland; ^4^ Cytogenetics Laboratory, Maria Sklodowska-Curie Institute - Oncology Center, Warsaw, Poland; ^5^ Department of Oncology, Poznan University of Medical Sciences, Poznan, Poland

**Keywords:** ovarian cancer, EMSY, polymorphism, gene expression, chemotherapy

## Abstract

EMSY, a BRCA2–associated protein, is amplified and overexpressed in various sporadic cancers. This is the first study assessing the clinical impact of its expression and polymorphisms on ovarian cancer (OvCa) outcome in the context of the chemotherapy regimen used. In 134 frozen OvCa samples, we assessed *EMSY* mRNA expression with Reverse Transcription-quantitative PCR, and also investigated the *EMSY* gene sequence using SSCP and/or PCR-sequencing. Clinical relevance of changes in *EMSY* mRNA expression and DNA sequence was evaluated in two subgroups treated with either taxane/platinum (TP, n=102) or platinum/cyclophosphamide (PC, n=32). High *EMSY* expression negatively affected overall survival (OS), disease-free survival (DFS) and sensitivity to treatment (PS) in the TP-treated subgroup (p-values: 0.001, 0.002 and 0.010, respectively). Accordingly, our OvCa cell line studies showed that the *EMSY* gene knockdown sensitized A2780 and IGROV1 cells to paclitaxel. Interestingly, *EMSY* mRNA expression in surviving cells was similar as in the control cells. Additionally, we identified 24 sequence alterations in the *EMSY* gene, including the previously undescribed: c.720G>C, p.(Lys240Asn); c.1860G>A, p.(Lys620Lys); c.246-76A>G; c.421+68A>C. In the PC-treated subgroup, a heterozygous genotype comprising five SNPs (rs4300410, rs3814711, rs4245443, rs2508740, rs2513523) negatively correlated with OS (p-value=0.009). The same SNPs exhibited adverse borderline associations with PS in the TP-treated subgroup. This is the first study providing evidence that high *EMSY* mRNA expression is a negative prognostic and predictive factor in OvCa patients treated with TP, and that the clinical outcome may hinge on certain SNPs in the *EMSY* gene as well.

## INTRODUCTION

Ovarian cancer is the most lethal malignancy of a female reproductive system. Due to the lack of specific symptoms and markers, most women are diagnosed in advanced stages of the disease. Despite a good initial response to chemotherapy, the majority of the patients develop a recurrent disease, and only approximately 30% of them survive 5 years [1]. Improvements in ovarian cancer treatment could be achieved by better understanding the molecular pathogenesis of this malignancy. This may also help identify molecular factors that determine success of the applied therapy and potentially allow for developing new methods of ovarian cancer treatment.

Currently, standard initial treatment of advanced stage ovarian carcinomas consists of maximal cytoreductive surgery and subsequent chemotherapy [2]. Platinum- and taxane-based regimens are routinely used as a first-line, postsurgical therapy in patients with FIGO stage II to IV disease. Mechanisms of action of the drugs used in these two regimens are different. Taxanes bind to ß-tubulin and stabilize microtubules by promoting their assembly and inhibiting disassembly [3]. This leads to a cell cycle arrest in the G2/M phase, cell division inhibition and apoptosis.

Platinum derivatives are crosslinking agents that damage DNA [4]. They create drug-DNA adducts that cause double-strand breaks in nucleic acid, resulting in apoptosis or necrosis of tumor cells. A platinum-caused DNA damage is repaired by different mechanisms, including homologous recombination (HR). *BRCA1* and *BRCA2* genes encode for two main tumor suppressor proteins of the HR DNA repair pathway. These genes are frequently mutated in familial ovarian cancer. In contrast, in sporadic ovarian tumors, both these genes are mutated in about 7% of cases only [5]. Nevertheless, they usually become inactive in a different way, such as the loss of heterozygosity or promoter hypermethylation [6]. It is suggested in the literature, that other proteins, e.g. EMSY, can impair the HR pathway by binding to BRCA2 and inhibiting its function.

EMSY is a nuclear protein that was first identified in complex with BRCA2. It consists of 1322 amino acids and is encoded by a gene (known as *EMSY* or *C11orf30*), located on chromosome 11 (cytoband 11q13.5) [7–9]. EMSY has an evolutionarily conserved ENT (EMSY N-terminal) domain, which directly binds to the BRCA2 protein and suppresses its transcriptional activity [10]. The EMSY protein is also capable of migrating to DNA repair sites, where it participates in a DNA damage response. When overexpressed, EMSY inactivates BRCA2, leading to a chromosomal instability and carcinogenesis [11–13]. The EMSY protein is also involved in the regulation of chromatin remodeling [14, 15] and in suppression of interferon (IFN)-stimulated genes in a BRCA2-dependent manner [16, 17]. Recently, novel BRCA2-independent functions of EMSY, involving interactions with different proteins and genes, have been found [18].

Amplification, and consequent overexpression, of *EMSY* mRNA and protein products has been detected in cancer cell lines from different organs [13] and in sporadic breast, ovarian, pancreatic, thyroid, and prostate tumors [13]. It was associated with poor outcome of the patients [19–22].

In ovarian cancers, amplification and/or over expression of *EMSY* was observed in 13-18% of cases [7, 23, 24]. The latter was found to promote growth, migration and tumorigenesis of ovarian cancer cells *in vitro* and *in vivo* [25]. Consistently, *EMSY* mRNA and protein levels were demonstrated to be up-regulated in ovarian cancer compared to a normal ovarian tissue [23].

The aim of this study was to investigate *EMSY* gene alterations and expression in ovarian cancer, and to evaluate their impact on survival and chemotherapy response.

## RESULTS

### *EMSY* genotyping

All 20 protein-coding exons of the *EMSY* gene, including intron/exon boundaries, were analyzed for DNA sequence variants in 134 non-consecutive ovarian carcinomas.

**Figure 1:**
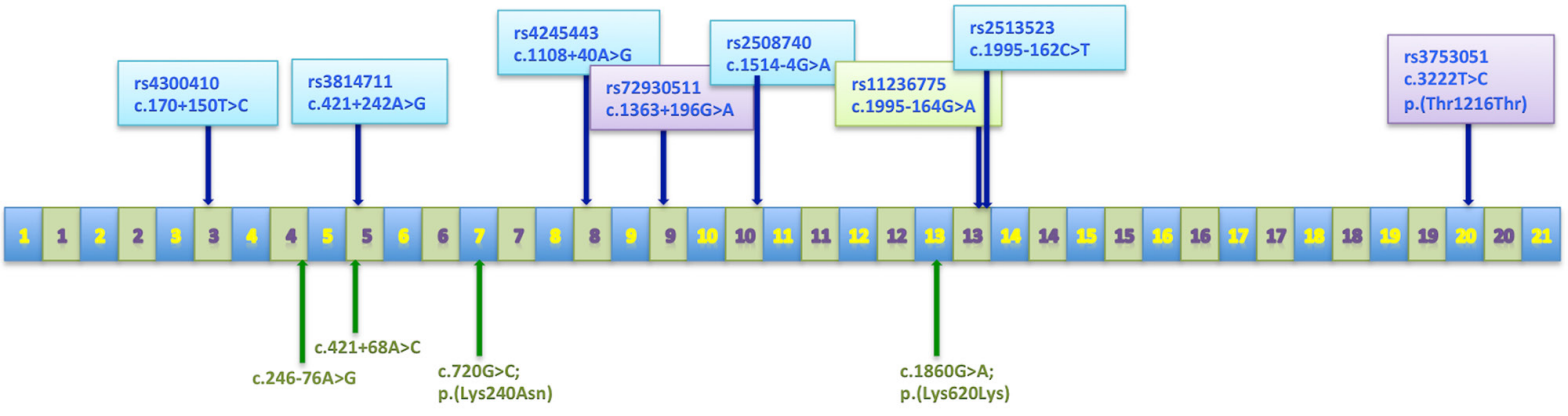
The *EMSY* gene organization Exons are represented by blue boxes and introns by green ones. Positions of polymorphisms are indicated by arrows. SNPs marked with various colors belong to different haplotypes. Novel sequence alterations are written in green type.

Altogether, we detected 24 sequence alterations (listed in Table 1). All of them were single-nucleotide substitutions. Twenty alterations were located in introns and only four were found in the protein-coding regions of the gene. Three of these exonic substitutions were synonymous, and one was of the missense type. Remarkably, we identified herein four genetic changes that have not been described before (Figure 1, Table 1). Each novel variant was detected once, in a single patient (0.7%), one was germline c.720G>C; p.(Lys240Asn), two were somatic (one in an exon and one in an intron). The last one (c.246-76A>G change) was of unknown origin, since there was no control tissue available.

**Table 1:** Sequence variants detected in the *EMSY* gene

No.	Location	Sequence variant	SNP ID
1	Exon VII	c.720G>C, p.(Lys240Asn)	novel, germline
2	Exon XIII	c.1860G>A, p.(Lys620Lys)	novel, somatic
3	Exon XIX	c.3222C>G, p.(Pro1074Pro)	rs35962163
4	Exon XX	c.3648T>C, p.(Thr1216Thr)	rs3753051
5	Intron III	c.170+150T>C	rs4300410
6	Intron IV	c.246-73T>A	rs34407750
7	Intron IV	c.246-76A>G	novel, na^*^
8	Intron V	c.421+68A>C	novel, somatic
9	Intron V	c.421+242A>G	rs3814711
10	Intron VII	c.831-102G>A	rs140211752
11	Intron VIII	c.1108+40A>G	rs4245443
12	Intron VIII	c.1109-60G>C	rs74680029
13	Intron IX	c.1363+120G>A	rs10899226
14	Intron IX	c.1363+196G>A	rs72930511
15	Intron IX	c.1363+234A>G	rs183707624
16	Intron IX	c.1363+291A>G	rs139270987
17	Intron X	c.1514-4G>A	rs2508740
18	Intron XI	c.1685-7T>C	rs199909771
19	Intron XI	c.1685-14C>T	rs11600501
20	Intron XIII	c.1995-152A>G	rs80237143
21	Intron XIII	c.1995-162C>T	rs2513523
22	Intron XIII	c.1995-164G>A	rs11236775
23	Intron XIV	c.2194+112T>C	rs566452599
24	Intron XX	c.3774+112T>C	rs11602123

^*^ na- origin of the change not analyzed due to the lack of a control tissue for this patient.

### Characteristics of the most common *EMSY* gene polymorphisms

Herein, we focused on eight polymorphisms with the highest allelic variability and the minor allele frequency (MAF) of over 15% (Figure 1). Those SNPs were genotyped in 134 ovarian carcinomas and in the A2780 and IGROV1 cell lines. The genotypes and their frequencies are presented in Table 2.

**Table 2:** Distribution and frequency of genotypes of the eight most common *EMSY* gene polymorphisms identified in this study

Polymorphism NCBI ID	Genotype				
	Tumors n (frequency)			Cell lines	
				A2780	IGROV1
rs4300410	C/C	C/T	T/T	C/T	C/C
	51 (38%)	66 (49%)	17 (13%)		
rs3814711	G/G	A/G	A/A	G/G	G/G
	55 (41%)	65 (49%)	14 (10%)		
rs4245443	G/G	A/G	A/A	G/G	G/G
	51 (38%)	67 (50%)	16 (12%)		
rs72930511	G/G	G/A	A/A	G/G	G/G
	65 (48%)	60 (45%)	9 (7%)		
rs2508740	A/A	A/G	G/G	A/A	A/A
	55 (41%)	66 (49%)	13 (10%)		
rs2513523	T/T	C/T	C/C	T/T	T/T
	46 (34%)	64 (48%)	24 (18%)		
rs11236775	G/G	A/G	A/A	G/G	A/A
	88 (66%)	37 (27%)	9 (7%)		
rs3753051	T/T	C/T	C/C	T/T	T/T
	63 (47%)	64 (48%)	7 (5%)		

**Figure 2:**
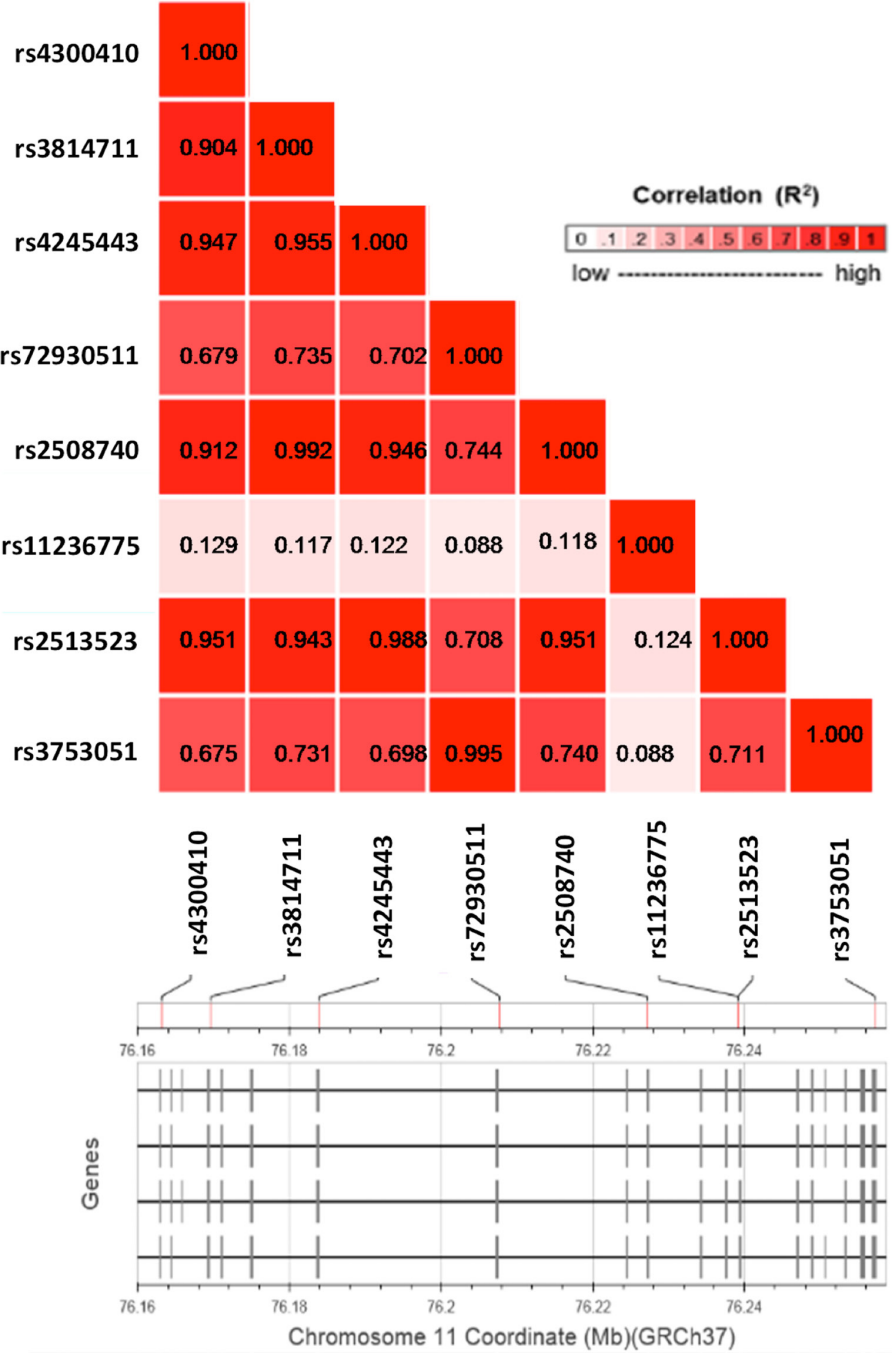
A linkage disequilibrium plot for the eight most common SNPs within the *EMSY* gene Pairwise LD (R^2^) is shown for each combination of SNPs.

The analysis of allele distribution revealed that some of the polymorphisms were linked. In order to confirm linkage disequilibrium (LD) of the eight most frequent SNPs, we performed an *in silico* analysis in a population of European ancestry with the use of a web-based application: LDlink (http://analysistools.nci.nih.gov/Ldlink/), which utilizes data from phase 3 of the 1000 Genomes Project. According to this bioinformatic tool, the SNPs, that we identified, can be aggregated in three groups with a very high LD (R^2^ > 0.9; Figure 2). The first group (LD5) consisted of 5 SNPs: rs4300410, rs3814711, rs4245443, rs2508740 and rs2513523. The second group (LD2) comprised two polymorphisms: rs72930511 and rs3753051. The last SNP, rs11236775, was not linked to the others (R^2^ < 0.13).

### *EMSY* mRNA expression

**Figure 3:**
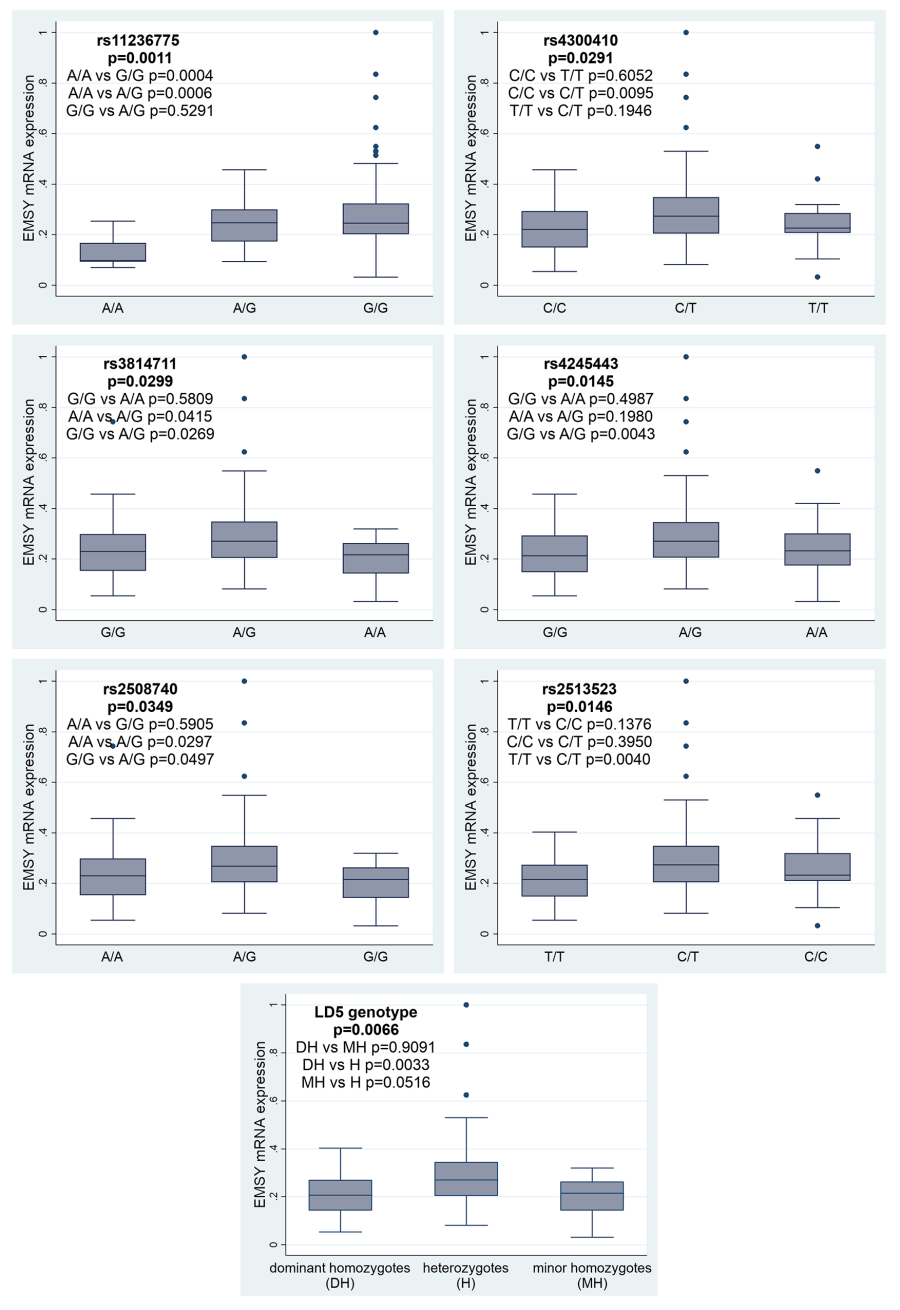
Associations between *EMSY* mRNA expression and six SNPs or the LD5 genotype in the same gene

Herein, we evaluated correlations between *EMSY* expression and the eight most common SNPs. All polymorphisms, except for those belonging to the LD2 group (rs3753051 and rs72930511), were significantly associated with the *EMSY* mRNA level. For the rs11236775 SNP, the lowest expression was observed in tumors with the A/A genotype, higher in heterozygotes A/G (p=0.0006, Figure 3), and the highest in homozygotes G/G (p=0.0004). There was no difference in *EMSY* mRNA expression between the G/G and A/G genotypes (p=0.5291). For the LD5 genotype, the highest level of *EMSY* mRNA was observed in heterozygotes (the p-value equaled 0.0066), while homozygotes had similarly low levels of mRNA expression. Consistently, we found the same regularity for all the SNPs forming this genotype with p-values ranging from 0.0145 to 0.0349 (Figure 3).

A statistical analysis using the Kruskal-Wallis test was performed to determine associations between the *EMSY* gene expression and a histological type, grade, clinical stage (FIGO) of a tumor and patient age. Serous tumors had significantly higher *EMSY* mRNA levels than the other histological types (p=0.0036). No other associations were identified.

### Evaluation of a clinical significance of genetic alterations found

Multivariate statistical analyses were performed to study associations of each particular SNP and *EMSY* mRNA expression with the clinical outcome in the entire group of ovarian cancer patients (n=134), and also in subgroups treated with either the taxane/platinum (TP, n=102) or platinum/cyclophosphamide (PC, n=32) regimen. In this analysis, a genotype with the shortest overall survival (OS) was always treated as a reference.

### Taxane/platinum-treated subgroup

#### Overall survival and disease-free survival

**Figure 4:**
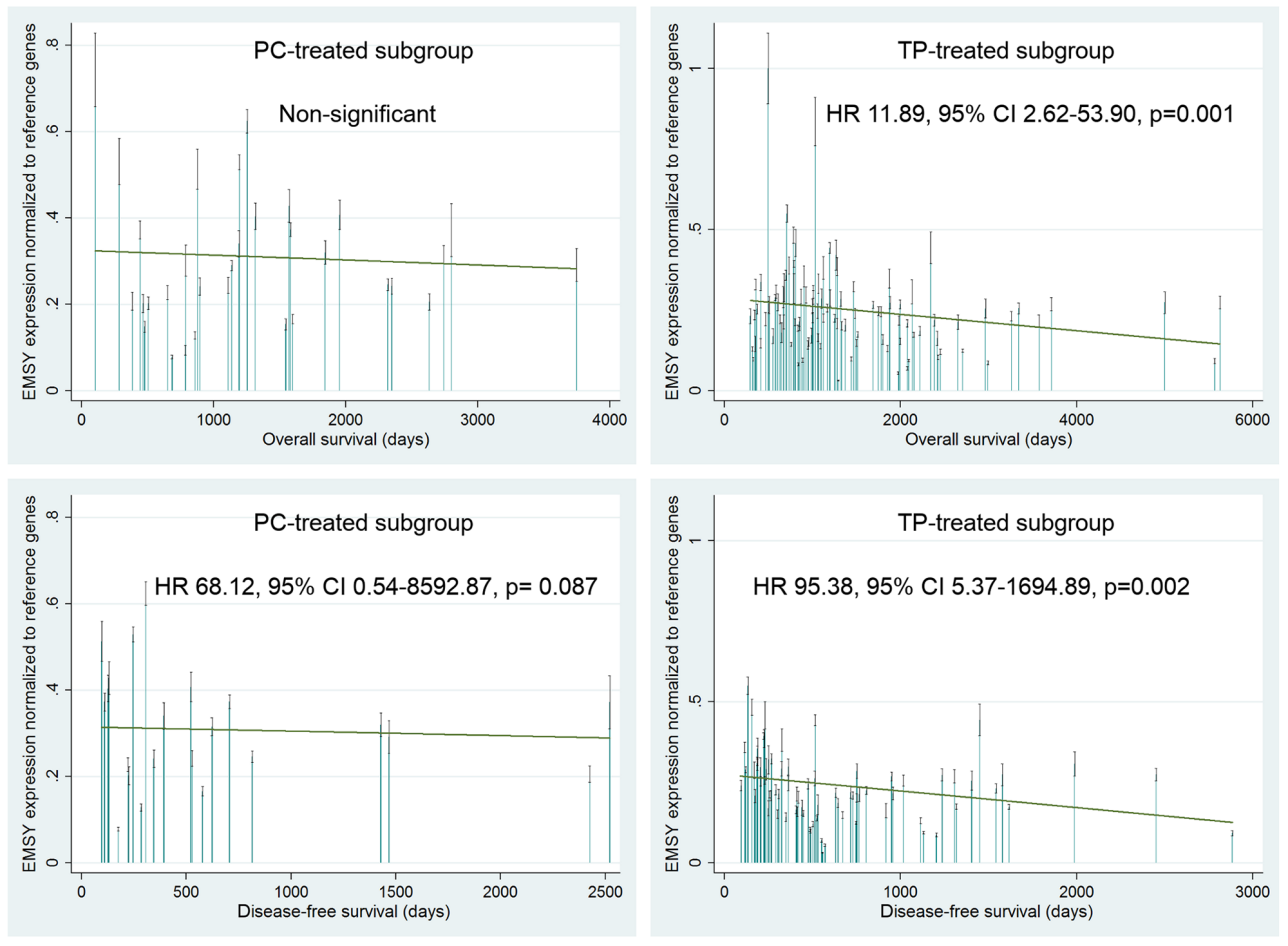
Evaluation of a prognostic value of the *EMSY* gene expression in the PC- and TP-treated subgroups of ovarian cancer patients A multivariate statistical analysis was performed using the Cox proportional hazards model. Green linear regression lines visualize trends in the expression change. Non-significant – a result with a p-value ≥0.1.

Overall survival (OS) and disease-free survival (DFS) were strongly associated with the *EMSY* gene expression in the TP-treated patients. A multivariate analysis revealed that elevated mRNA levels were a negative prognostic factor, related to increased risks of death and relapse (p=0.001 and p=0.002 respectively, Table 3, Figure 4). The same associations were found in the joined TP- and PC-treated group (p=0.003 for OS and p=0.001 for DFS, Table 3). Noteworthy, the OS-related results were significant at a lower level of statistical significance in the joined group, despite its bigger size.

**Table 3:** The multivariate statistical analysis (Cox proportional hazards model) of a prognostic value of *EMSY* mRNA expression and SNPs in ovarian cancer patients treated with either the TP or PC regimen

		Overall survival						Disease-free survival					
		PC+TP regimen (117/134) ^a^		TP regimen (86/102)^a^		PC regimen (31/32)^a^		PC+TP regimen (83/96)^a^		TP regimen (63/74)^a^		PC regimen (20/22)^a^	
Variable name		Median (n)	HR [95% CI] p	Median (n)	HR [95% CI] p	Median (n)	HR [95% CI] p	Median (n)	HR [95% CI] p	Median (n)	HR [95% CI] p	Median (n)	HR [95% CI] p
**expression**													
high vs low			8.04 [1.99-32.43] **0.003**		11.89 [2.62-53.90] **0.001**		NS		38.93 [4.40-344.27] **0.001**		95.38 [5.37-1694.89] **0.002**		68.12 [0.54-8592.87] 0.087
**rs11236775**													
A/A		786 (9)	1	770.5 (6)	1	786 (3)	1	528 (6)	1	528 (4)	1	377 (2)	1
A/G		1009 (37)	NS	1010 (30)	NS	897 (7)	NS	256 (25)	NS	256 (21)	NS	284 (4)	NS
G/G		1256 (89)	NS	1275.5 (66)	NS	1194.5 (22)	0.26 [0.06-1.25] 0.093	515 (66)	NS	514 (49)	NS	458 (16)	NS
**LD5 haplotype**													
dominant homozygotes		1010 (44)	1	1040 (34)	1	875.5 (10)	1	444.5 (30)	1	528 (24)	1	230.5 (6)	1
heretozygotes		1138 (59)	NS	1085.5 (40)	NS	1196 (19)	0.19 [0.05-0.66] **0.009**	414 (41)	NS	414 (27)	NS	458 (14)	NS
minor homozygotes		1295 (13)	NS	1288.5 (12)	NS	2742 (1)	0.03 [0.0008-0.86] 0.041	500.5 (12)	NS	487 (11)	NS	623 (1)	0.04 [0.0008-1.74] 0.094
**LD5 haplotype**	**rs4300410**												
	C/C	1011 (51)	1	1040 (40)	1	897 (11)	1	256 (36)	1	305.5 (29)	1	127 (7)	1
	C/T	1138 (67)	NS	1085.5 (46)	NS	1194.5 (20)	0.35 [0.13-0.97] 0.043	247 (46)	NS	414 (31)	NS	236.5 (14)	NS
	T/T	1295 (17)	NS	1288.5 (16)	NS	2742 (1)	0.04 [0.001-1.23] 0.065	476 (15)	NS	413 (14)	NS	623 (1)	NS
	**rs3814711**												
	G/G	1011 (55)	1	1069 (43)	1	878.5 (12)	1	444.5 (38)	1	525 (31)	1	285 (7)	1
	G/A	1165.5 (66)	NS	1085.5 (46)	NS	1196 (19)	0.28 [0.10-0.76] **0.013**	445 (46)	NS	414 (31)	NS	458 (14)	NS
	A/A	1305.5 (14)	NS	1295 (13)	NS	2742 (1)	0.03 [0.0008-0.85] 0.041	514 (13)	NS	500.5 (12)	NS	623 (1)	NS
	**rs4245443**												
	G/G	1011 (51)	1	1040 (40)	1	897 (11)	1	469 (36)	1	525 (29)	1	285 (7)	1
	A/G	1153 (68)	NS	1105 (47)	NS	1194.5 (20)	0.35 [0.13-0.97] 0.043	414 (47)	NS	413 (32)	NS	458 (14)	NS
	A/A	1288.5 (16)	NS	1282 (15)	NS	2742 (1)	0.04 [0.001-1.23] 0.065	481.5 (14)	NS	476 (13)	NS	623 (1)	NS
	**rs2508740**												
	A/A	1011 (55)	1	1069 (43)	1	878.5 (12)	1	444.5 (38)	1	525 (31)	1	285 (7)	1
	A/G	1193 (67)	NS	1105 (47)	NS	1196 (19)	0.28 [0.10-0.76] **0.013**	476 (47)	NS	445 (33)	NS	458 (14)	NS
	G/G	1295 (13)	NS	1288.5 (12)	NS	2742 (1)	0.03 [0.0008-0.85] 0.041	500.5 (12)	NS	487 (11)	NS	623 (1)	NS
	**rs2513523**												
	T/T	1010 (46)	1	1040 (36)	1	878.5 (10)	1	430.5 (32)	1	486.5 (26)	1	230.5 (6)	1
	C/T	1193 (65)	NS	1115 (44)	NS	1194.5 (20)	0.26 [0.08-0.86] 0.028	490 (45)	NS	452 (30)	NS	458 (14)	NS
	C/C	1288.5 (24)	NS	1275.5 (22)	NS	2162.5 (2)	0.11 [0.01-1.18] 0.069	500.5 (20)	NS	481.5 (18)	NS	665 (2)	NS

^a^) Values before and after a slash (/) stand for the number of completed observations versus all observations. Only the results with p-values < 0.1 are shown, and those with p-values < (0.05/3≈0.02) (after the Bonferroni correction) are highlighted in bold type. HR – hazard ratio; CI – confidence interval; n – number of cases; NS – non-significant result (p ≥ 0.1).

We did not find any statistically significant differences in OS and DFS depending on *EMSY* polymorphisms in the TP-treated subgroup (Table 3).

#### Sensitivity to therapy and complete remission

**Figure 5:**
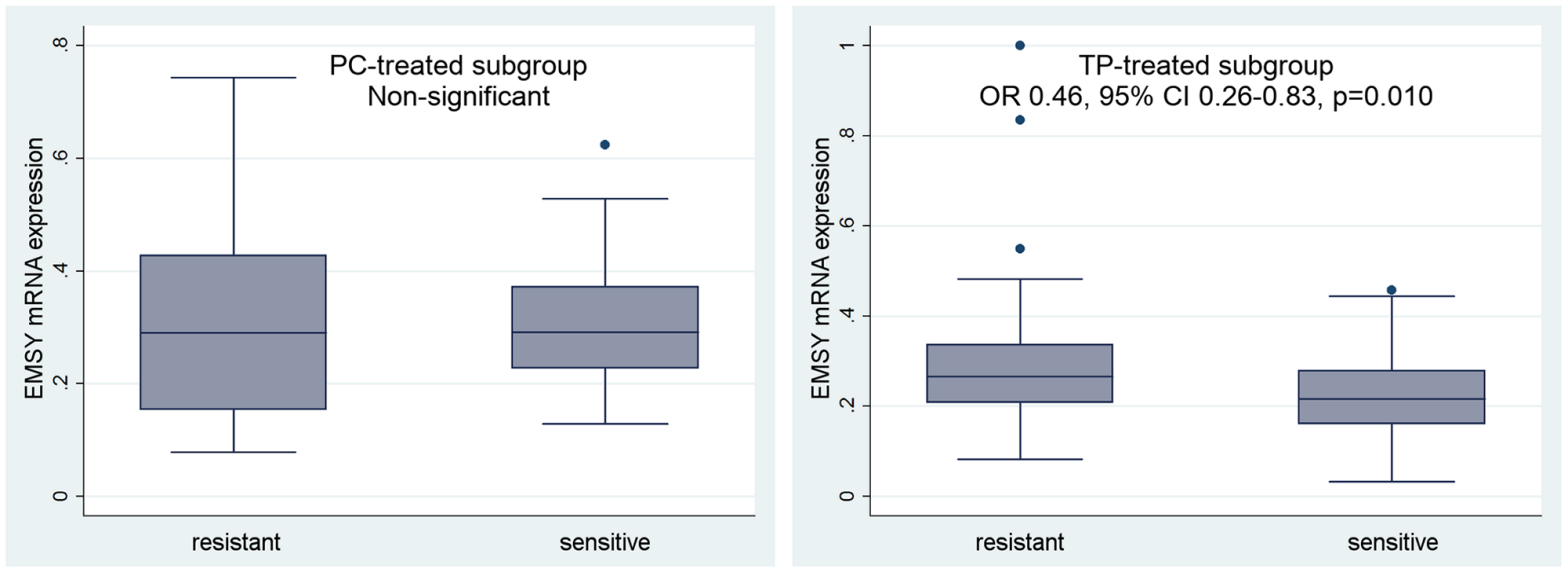
Evaluation of a predictive value of the *EMSY* gene expression in the PC- and TP-treated subgroups of ovarian cancer patients We analyzed the relationship between *EMSY* expression and sensitivity to treatment (PS). According to the multivariate logistic regression model, this regularity was statistically significant only in the TP-treated subgroup. Non-significant – a result with a p-value ≥0.1.

In the TP-treated subgroup, sensitivity to therapy (PS) was negatively associated with *EMSY* gene expression and likely also with the polymorphisms belonging to the LD5 genotype (borderline significance). Ovarian cancer patients with higher *EMSY* mRNA expression in tumors exhibited lower sensitivity to the TP chemotherapy (p=0.010, Table 4, Figure 5). Similarly, the presence of the heterozygous LD5 genotype, which correlated with higher *EMSY* mRNA levels, decreased the probability of treatment sensitivity by about 60% (the p-values were on the border of statistical significance, equaling 0.079 for the entire genotype, and ranging from 0.047 to 0.068 for individual SNPs, Table 4).

**Table 4:** The multivariate statistical analysis (logistic regression model) of a predictive value of *EMSY* mRNA expression and SNPs in ovarian cancer patients treated with either the TP or PC regimen

Variable name		Platinum sensitivity					
		PC+TP regimen (84/134)^a^		TP regimen (67/102)^a^		PC regimen (17/32)^a^	
		x/y	OR [95% CI] p	x/y	OR [95% CI] p	x/y	OR [95% CI] p
**expression**							
high vs low			0.59 [0.39-0.91] **0.017**		0.46 [0.26-0.83] **0.010**		NS
**rs11236775**							
A/A		5/9 (56%)	1	4/6 (67%)	1	1/3 (33%)	1
A/G		22/37 (60%)	NS	19/30 (63%)	NS	3/7 (43%)	NS
G/G		57/88 (65%)	NS	44/66 (67%)	NS	13/22 (59%)	NS
**LD5 haplotype**							
dominant homozygotes		27/44 (61%)	1	24/34 (71%)	1	3/10 (30%)	1
heretozygotes		34/59 (58%)	NS	22/40 (55%)	0.36 [0.12-1.13] 0.079	12/19 (63%)	NS
minor homozygotes		11/13 (85%)	NS	10/12 (83%)	NS	1/1 (100%)	NS
**LD5 haplotype**	**rs4300410**						
	C/C	33/51 (65%)	1	29/40 (73%)	1	4/11 (36%)	1
	C/T	38/66 (58%)	NS	26/46 (57%)	0.36 [0.13-1.01] 0.052	12/20 (60%)	NS
	T/T	13/17 (77%)	NS	12/16 (75%)	NS	1/1 (100%)	NS
	**rs3814711**						
	G/G	35/55 (64%)	1	31/43 (72%)	1	4/12 (33%)	1
	G/A	37/65 (57%)	NS	25/46 (54%)	0.36 [0.13-0.99] 0.047	12/19 (63%)	NS
	A/A	12/14 (86%)	NS	11/13 (85%)	NS	1/1 (100%)	NS
	**rs4245443**						
	G/G	33/51 (65%)	1	29/40 (73%)	1	4/11 (36%)	1
	A/G	39/67 (58%)	NS	27/47 (57%)	0.39 [0.14-1.07] 0.068	12/20 (60%)	NS
	A/A	12/16 (75%)	NS	11/15 (73%)	NS	1/1 (100%)	NS
	**rs2508740**						
	A/A	35/55 (64%)	1	31/43 (72%)	1	4/12 (33%)	1
	A/G	38/66 (58%)	NS	26/47 (55%)	0.38 [0.14-1.03] 0.058	12/19 (63%)	NS
	G/G	11/13 (85%)	NS	10/12 (83%)	NS	1/1 (100%)	NS
	**rs2513523**						
	T/T	29/46 (63%)	1	26/36 (72%)	1	3/10 (30%)	1
	C/T	37/64 (58%)	NS	25/44 (57%)	0.33 [0.11-0.98] 0.047	12/20 (60%)	NS
	C/C	18/24 (75%)	NS	16/22 (73%)	NS	2/2 (100%)	NS

^a^ Values before and after a slash (/) stand for the number of tumors positively responding to the treatment (x) versus all tumors (y). Only the results with p-values < 0.1 are shown, and those with p-values < (0.05/3≈0.02) (after the Bonferroni correction) are highlighted in bold type. OR – odds ratio; CI – confidence interval; n – number of cases; NS – non-significant result (p ≥ 0.1).

We did not find any statistically significant associations between PS and polymorphisms forming the LD2 genotype (rs72930511 and rs3753051, data not shown) or rs11236775 (Table 4).

We also identified a borderline correlation between higher *EMSY* gene expression and lower chance for complete remission (CR) (OR 0.57, 95% CI 0.33-0.97, p=0.040) in the TP-treated patients. No associations were found between CR and any polymorphism analyzed herein.

### Platinum/cyclophosphamide-treated subgroup

#### Overall survival and disease-free survival

In the PC-treated patients, overall survival (OS) was negatively associated with the SNPs belonging to the LD5 genotype. We also observed a similar trend for the rs11236775 polymorphism, being on the border of statistical significance.

Polymorphisms aggregated in the LD5 genotype were significantly related to OS. Patients with a heterozygous genotype, that correlated with higher *EMSY* gene expression (Figure 3), had the risk of death decreased by approximately 80% compared to those with the dominant homozygous genotype. The p-values equaled 0.009 for OS for the entire LD5 genotype, and ranged from 0.013 to 0.043 for individual SNPs (Table 3). The same regularity was revealed in patients with minor homozygotes in the analyzed loci. However, since the relevant group comprised one or two specimens only, the result has to be interpreted with caution (Table 3).

We found no relationship between DFS and any variable in the PC-treated subgroup (Table 3). However, non-significant associations were observed between down-regulated *EMSY* mRNA expression (p=0.087), or the presence of minor homozygotes in the LD5 genotype (p=0.094), and the decreased risk of relapse.

#### Sensitivity to therapy and complete remission

In the PC-treated subgroup of patients, no associations were found with respect to the response to chemotherapy (CR and PS).

### Data bootstrapping and cross-validation of Cox and logistic regression models

We also performed a cross-validation of our results with multiple rounds of bootstrapping (with replacement) of the original data set, and subsequent evaluation of discriminating abilities of each model in both the original and bootstrapped data sets. Afterwards, performances of the models were assessed by comparing their areas under curves (AUCs) before and after cross-validation (please, see the ROC and AUC plots presented in Supplementary Figures 1-4). It is noteworthy that all AUC values, even after cross-validation in the small PC-treated subgroup, were still higher than 50 % (the AUC value of a null model), which proved that the models were not overtrained, and their performance was good, regardless of the data set used.

### Time trend analysis

**Figure 6:**
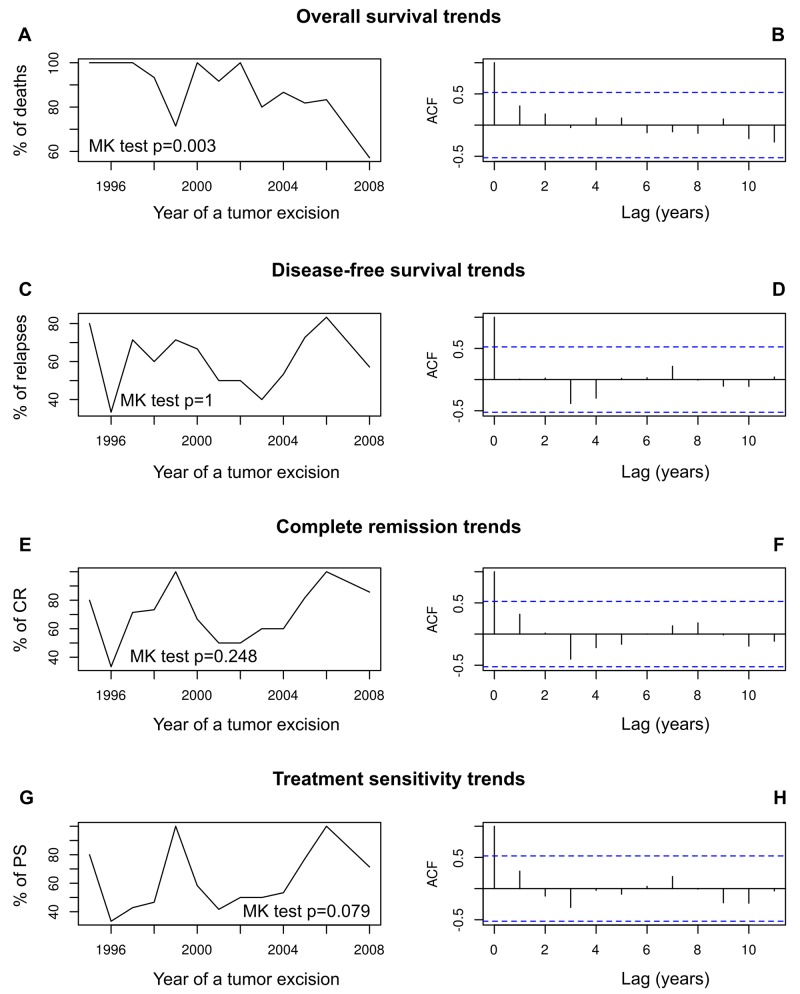
The analysis of prognostic and predictive time trends in our group of ovarian cancer patients These patients underwent their first surgical treatment in years 1995–2008. Time trends in overall survival **(A, B)**; disease-free survival **(C, D)**; complete remission **(E, F)**; sensitivity to chemotherapy **(G, H)**. The trends are shown as a trend line of death, relapse, CR and PS frequencies, respectively, supplemented with the results of Mann-Kendall homogeneity test, and supported with autocorrelation function (ACF) plots.

Our analysis of time trends revealed that the frequency of ovarian cancer-related death significantly diminished in a time frame of this study (Mann-Kendall test p = 0.003, Figure 6A–6B). No time trends were found for the frequencies of recurrence, complete remission or sensitivity to chemotherapy (Figure 6C–6H).

### Cell line studies

**Figure 7:**
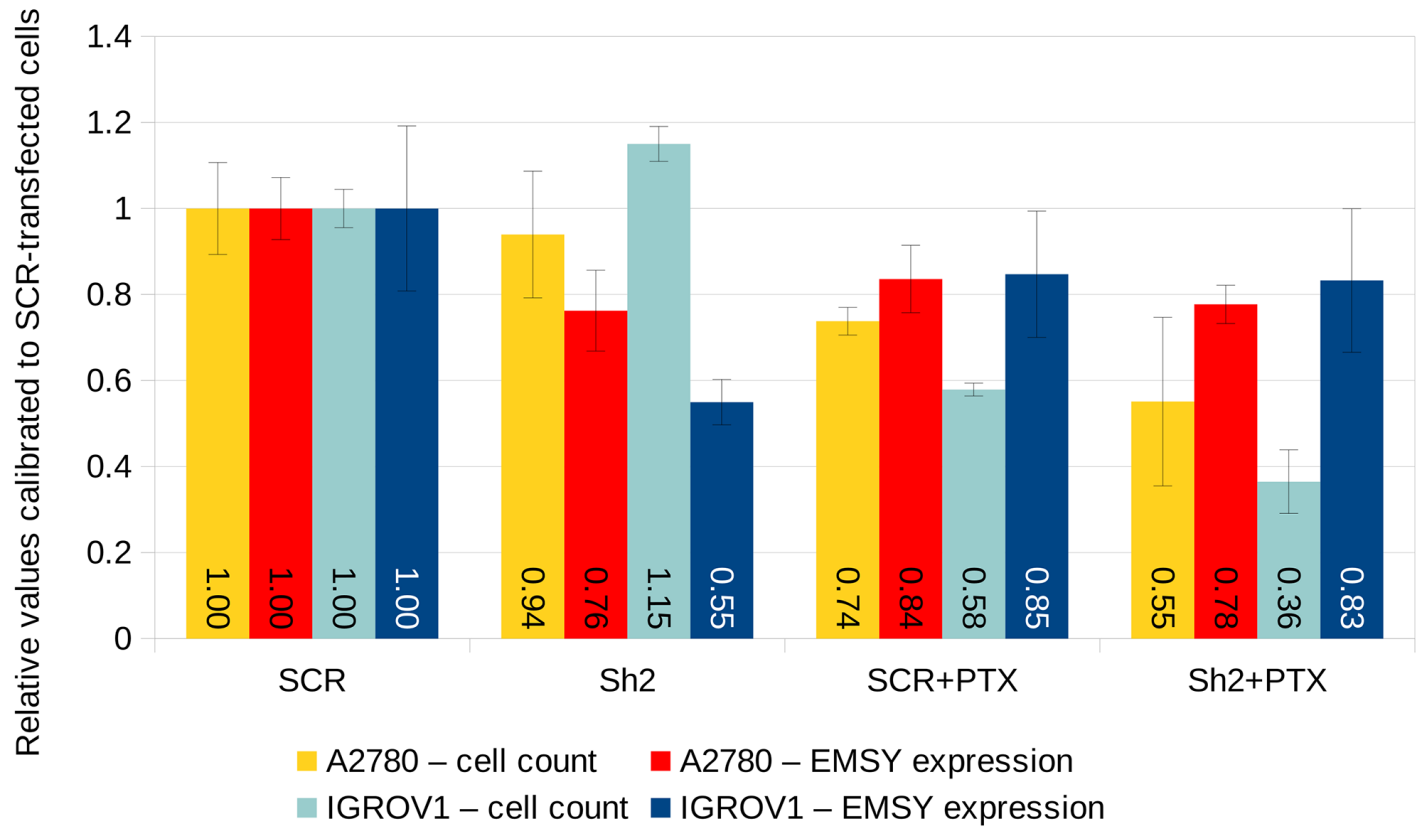
shRNA-mediated knockdown of *EMSY* mRNA expression in A2780 and IGROV1 ovarian cancer cell lines The *EMSY* gene was transiently knocked down with the Sh2 shRNA cloned into the pGFP-B-RS vector. The same vector harboring the SCR (scrambled) shRNA was used as a negative control. *EMSY* expression levels were normalized to three reference genes (*HGPRT*, *PPIA* and *GUSB*) and calibrated to SCR-transfected samples not treated with paclitaxel (PTX).

In order to elucidate whether the expression of *EMSY* mRNA affects ovarian tumors’ response to taxanes (which was observed in clinical samples), we carried out additional *in vitro* studies using two ovarian cancer cell lines A2780 and IGROV1. Initially, both cell lines were cultured with different concentrations of paclitaxel to assess the half maximal inhibitory concentration (IC_50_) of the drug. In compliance with the aforementioned results on clinical samples, the sensitivity of both cell lines to paclitaxel increased significantly after shRNA-mediated knockdown of *EMSY* mRNA. This effect was stronger in IGROV1 than in A2780 cells (Figure 7). Ultimately, 8 nM concentration of paclitaxel was applied to both cell lines, which was an approximate IC_50_ dose after the *EMSY* mRNA knockdown (Figure 7). As to the knockdown efficiency, *EMSY* mRNA levels diminished by 24% and 45% in A2780 and IGROV1 cells, respectively. However, given the transfection rate of about 50% for both cell lines, the real efficacy was ∼48% and ∼90%, respectively. Although the survival rate of the paclitaxel-treated cells was significantly lower after shRNA-mediated silencing of the *EMSY* gene, it is noteworthy that *EMSY* mRNA expression in surviving cells was comparable, regardless of the cell line and a type of the construct (silencing/control) used (Figure 7).

## DISCUSSION

In this study, we aimed to analyze expression and genetic alterations in the *EMSY* gene, and to evaluate their potential associations with clinical outcome of ovarian cancer patients. Our results revealed that changes in *EMSY* expression may influence cancer prognosis and significantly affect tumor response to chemotherapy. However, the latter seems to depend on the regimen applied.

To date, EMSY was regarded as a factor potentially influencing clinical outcome in cancer patients treated with DNA damaging agents [12, 26]. This notion was based on its involvement in BRCA2 regulation and the role it plays in DNA repair. Herein, we show for the first time that EMSY may affect the therapy with anti-microtubule drugs, as well. Our analysis of *EMSY* mRNA levels in patients treated with taxane/platinum compounds revealed that higher levels of *EMSY* mRNA accompanied a decreased drug-sensitivity, shortened the time to relapse and increased the risk of death. Similar associations, though detectable at a lower level of statistical significance, were found when the PC- and TP-treated subgroups were analyzed together.

Remarkably, our cell line experiments seem to support the results obtained in clinical samples, as higher *EMSY* mRNA expression was correlated with lower sensitivity to paclitaxel. This effect was stronger in IGROV1 than in A2780 cells. The differential response to the drug may be related to different genetic aberrations harbored by each of the cell lines [27, 28]. Interestingly, *EMSY* mRNA levels in the surviving cells were comparable, regardless of the cell line and a type of the construct (silencing/control) used. Such an effect seems to imply that paclitaxel selectively killed the cells with lower expression of *EMSY* mRNA. This may support our clinical results, and simultaneously suggests a pivotal role of *EMSY* expression in determining chemoresistance of ovarian cancer to taxanes.

To the best of our knowledge, the *EMSY* gene has not been investigated so far in context of the antimitotic therapy in any neoplasm. There is only one scientific report available showing that *EMSY* amplification, and its resultant overexpression, was linked to poor prognosis in ovarian cancer patients [29]. This study seems to support our results, giving another evidence for a conceivable involvement of EMSY in determination of the clinical outcome when the taxane-based regimen is used.

The way that EMSY impairs therapeutic effects of taxanes and/or negatively impacts cellular toxicity may hinge on its involvement in the gene expression regulation. One of the targets is small non-coding RNA – miR-31. The EMSY protein may directly bind to the miR-31 promoter which results in a decreased expression of this microRNA [30, 31]. miR-31 was proven to play an antimetastatic role in the cell and its down-regulation promotes development of breast and ovarian cancers [32–34]. In breast cancer cell lines, it was shown that overexpression of *EMSY* reduced the level of miR-31, and concomitantly increased expression of its target genes, thus inducing invasiveness and metastatic potential of the cells [30, 31]. Moreover, decreased miR-31 levels were found in taxane-resistant ovarian cancer cell lines and tissues [35, 36]. This chemoresistance can be abrogated by enforced expression of miR-31. Further exploration of the mechanism responsible for miR-31-dependent lack of sensitivity to taxanes resulted in identification of two proteins, MET and STMN1 (stathmin 1), that influence toxicity of the drug [35, 36]. Both of them are upregulated in taxane-resistant ovarian cancer cells due to low levels of miR-31. The oncogene *MET* encodes a receptor tyrosine kinase that activates pathways involved in cell survival and migration [37]. In a mouse xenograft model, it was shown that overexpression of miR-31, as well as supplementary therapy with a MET inhibitor could overcome chemoresistance, decrease a size of the tumor and improve overall survival of taxane-treated mice [35]. The other molecule, STMN1, is a microtubule-associated protein. In contrast to taxol, STMN1 destabilizes microtubule filaments by preventing their assembly and promoting disassembly [36]. In step with this mechanism of action, it was shown that high stathmin 1 expression was an adverse prognostic factor in ovarian cancer patients who received taxane-platinum combination chemotherapy. On the contrary, such an effect was not observed in those patients who were administered with the platinum-based regimen only [38].

It has to be noted that initially a larger fragment of chromosome 11 (11q13) was found to be amplified in many types of cancer including ovarian tumors [19, 29]. Later, numerous genes, such as *CCND1*, *EMS1*, *PAK1*, *GA2*, *RSF1*, *EMSY* and others, were mapped to this locus. Since then, it has been broadly studied and discussed in the literature on which of these genes is responsible for the clinical effect observed [29, 39]. In the study by Brown et al., [29], *EMSY* and *RSF1* were the only genes at this locus proven to be associated with significantly worse outcome. In their cohort of ovarian tumors analyzed, *EMSY* was more frequently amplified than *RSF1* (16% vs 12%), yet both genes were co-amplified in about 65% of cases. Recently, it was demonstrated that overexpression of the *RSF1* gene contributed to paclitaxel resistance [40]. Therefore, it is likely that either amplification or overexpression of *EMSY* and *RSF1* may contribute to taxane resistance in ovarian cancer.

EMSY was first identified as a BRCA2-binding partner and it is believed to play a role in homologous recombination-mediated repair of DNA double-strand breaks [7]. It co-localizes with γH2AX foci, a marker of double-strand breaks, after ionizing irradiation. Overexpression of *EMSY* elicits a “chromosome instability phenotype” similar to that observed in BRCA2-deficient cells [7, 11, 41]. Consistently, amplification of the *EMSY* gene has been proposed to mimic the *BRCA2*-mutant phenotype which might be a mechanism of BRCA2 pathway inactivation and consequent sensitization of cancer cells to DNA damaging drugs. Such an effect was observed in some studies [26, 42]. On the other hand, Wilkerson and colleagues questioned the role of EMSY in DNA repair, and its ability to affect sensitivity to DNA-damaging agents or PARP inhibitors [13]. In their study, cell lines with or without *EMSY* amplification had similar sensitivity to cisplatin and olaparib. On the contrary, the cell lines with *BRCA1* or *BRCA2* mutations exhibited higher sensitivity to the drugs compared to those with the wild type *BRCA1/2* genes. Moreover, siRNA-mediated silencing of *EMSY* expression in cell lines with *EMSY* amplification had no effect on their sensitivity to cisplatin and olaparib. Noteworthy, there is also one recent study that suggested that EMSY participation in DNA repair might be BRCA2-independent [41]. Considering all these discrepancies, the role of *EMSY* in DNA repair and cancer therapy still remains unclear and requires further investigation. In our research, we did not find any statistically significant associations between *EMSY* mRNA levels and the outcome of patients treated with DNA-damaging agents only. This might be related to the small size of our PC-treated subgroup (32 patients). Nevertheless, the same analysis of *EMSY* expression in all the cases (administered with either TP or PC) revealed lower statistical significance compared to the sole TP-treated subgroup. This may suggest a strongly decreased or even opposed clinical effect in PC-treated patients, and seems to be supported by one of our multivariate Cox regression models for the LD5 genotype. The model revealed that heterozygotes (earlier proved to be correlated with high *EMSY* mRNA expression) were also associated with a favorable clinical outcome (OS). It is worth noting that this regularity was found only in the PC-treated subgroup of patients.

Nowadays, platinum-based compounds are not administered to ovarian cancer patients as the first-line chemotherapy. DNA-damaging agents only may, however, be used as second- and further lines of treatment. Furthermore, the clinical associations that we report herein are conceivably potentially applicable to other malignancies treated with DNA damaging agents, such as lung cancers, testicular cancers, melanomas, myelomas and lymphomas [43].

Herein, we also found an association between *EMSY* gene polymorphisms and outcome in ovarian cancer patients. Our study is the first one that investigated this problem. Previous research have focused mostly on the relationship between *EMSY* gene variants and the risk of developing cancer. Polymorphisms, including rs4245443, rs2508740, rs11600501, rs3753051, were analyzed in breast and ovarian cancers [44, 45], and there was no evidence that any of these polymorphisms influenced the risk of the disease in white British and Finnish populations. *EMSY* sequence variants have also been analyzed in prostate cancer in Finnish patients and healthy controls [46, 47]. The authors paid attention to a clinical significance of the detected alterations. A rare intronic variant, rs200331695, was found to increase the risk of familial prostate cancer and to contribute to the aggressive course of both familial and sporadic variants of the disease [46]. Moreover, the analysis of segregation of the alleles in two families with prostate cancer revealed that the same unfavorable polymorphic variant was also present in a sister of one of the probands, who developed breast cancer [46]. Other two intronic *EMSY* SNPs, rs10899221 and 72944758, were linked to the prostate cancer risk, as well. None of them, however, was shown to be related to patient outcome [47].

In our group of ovarian cancer patients, five of the eight polymorphisms analyzed seemed to have a prognostic and/or predictive relevance depending on a chemotherapeutic regimen used. Polymorphisms belonging to the LD5 genotype affected OS in the PC-treated patients and PS in those treated with TP. However, the latter association was identified on the border of statistical significance. All of the SNPs are located in introns, and the way they affect clinical outcome remains unclear. The underlying molecular mechanism may potentially involve alterations in *EMSY* pre-mRNA processing, especially that one of the polymorphisms forming the LD5 genotype (rs2508740) is located in a proximity of an intron/exon boundary. In step with this assumption, there are as many as 23 different mRNAs in the AceView database which have been reported as splice variants of the human *EMSY* gene [48]. Furthermore, by using antibodies targeting distinct protein fragments, the EMSY protein was detected in either the nucleus only or in both the nucleus and cytoplasm [19]. This may suggest the existence of various protein isoforms in a cell that probably act in a different way during cancer development.

Other potential mechanisms of clinical significance of *EMSY* polymorphisms should also be considered. In agreement with this hypothesis, some genetic changes within the *EMSY* locus are bound to atopic diseases like asthma or allergic sensitization [49]. Moreover, it has recently been proven that EMSY may influence the immune system function by regulating the IFN signaling pathway, thus potentially affecting both tumorigenesis and the antitumor immune response [17, 50]. On the other hand, the prognostic significance of the SNPs we described herein may also be caused by their linkage to another functional polymorphism, currently unidentified.

The limitation of our study is that genetic variation of the *EMSY* gene had to be evaluated in tumors due to the lack of a normal tissue for some cases. This might have potentially led to erroneous conclusions due to somatic alterations in cancer, e.g., amplification of the *EMSY* gene. Therefore, the results of our polymorphism analysis should be interpreted with caution.

Finally, we have found 4 novel sequence variants in the *EMSY* gene. Their connection to cancer risk and clinical outcome remains unclear. One of the changes was a germline missense substitution in exon 7, c.720G>C; p.(Lys240Asn). This alteration is located within the first 478 amino acids of the EMSY protein, crucial for its interaction with BRCA2 [9]. Thus, the involvement of this genetic change in cancer development cannot be excluded. Nevertheless, further studies on bigger cohorts of patients are needed to unequivocally elucidate its clinical meaning.

Considering the retrospective character of our study, with clinical samples being collected for 14 years, we decided to perform a time-trend analysis to make sure that the risks of death, relapse, as well as the chances for complete remission and treatment sensitivity do not depend on sample collection time. This analysis revealed that only the risk of death significantly changed in the time frame of our study. It is likely that introduction of the taxane-based treatment could have been one of the factors that contributed to a significant decrease in death frequency that we observed in our group of patients. Higher overall effectiveness of this regimen was the main reason why taxanes were recommended as the first-line chemotherapy of ovarian cancer patients [51].

Our study is the first to provide evidence that high *EMSY* expression is a negative prognostic and predictive factor in ovarian cancer patients treated with TP. We also demonstrated that the clinical meaning of SNPs in the *EMSY* gene depended on the chemotherapy regimen used. Thus, the *EMSY* mRNA expression could potentially be utilized as a marker of cancer prognosis and its response to chemotherapy. In addition, the analysis of SNPs within this gene could be useful during selection of the most effective method of treatment for each patient.

## MATERIALS AND METHODS

### Ovarian cancer patients and tumors

The study was performed on 134 non-consecutive samples from patients from central Poland. The patients were all diagnosed with epithelial ovarian carcinoma. Frozen tumor fragments and blood samples (used only for confirmation of a germline origin of detected genetic alterations) were collected in two hospitals in Warsaw (Maria Sklodowska-Curie Institute - Oncology Center and Brodnowski Hospital) between the years 1995 and 2008. The material used in this research was carefully selected out of 400 cases by at least two clinicians for patient selection to meet the following criteria: no chemotherapy before staging laparotomy, adequate staging procedure, International Federation of Gynecologist and Obstetricians (FIGO) stage IIB to IV [52], tumor tissue from the first laparotomy available. All tumors were uniformly reviewed histopathologically, and classified histologically according to the World Health Organization [53], and graded in a four-grade scale, in compliance with the standards given by Barber et al. [54].

All the specimens utilized in this study had detailed clinical information available, including the residual tumor size and follow-up data. The patients belonged to two subgroups: treated with either taxanes and cisplatin/carboplatin (TP regimen, n=102) or cisplatin and cyclophosphamide (PC regimen, n=32). A clinicopathological characteristics of the patients and tumors is presented in Table 5.

**Table 5:** Clinicopathological characteristics of patients and tumors

	TP subgroup (n=102)	PC subgroup (n=32)
**Age (years)**		
Range (median)	20-79 (53)	34-68 (54)
**Histological type**		
Serous	78 (76.5%)	31 (96.9%)
Endometrioid	4 (3.9%)	0 (0%)
Undifferentiated	8 (7.8%)	0 (0%)
Other types	12 (11.8%)	1 (3.1%)
**Histological grade**		
G1+G2	14 (13.7%)	4 (12.5%)
G3	59 (57.8%)	18 (56.3%)
G4	29 (28.4%)	10 (31.2%)
**Clinical stage (FIGO)**		
IIB, IIC	3 (2.9%)	0 (0.0%)
IIIA, IIIB	9 (8.8%)	6 (18.7%)
IIIC	83 (81.4%)	22 (68.8%)
IV	7 (6.9%)	4 (12.5%)
**Residual tumor size**		
0 cm	25 (24.5%)	8 (25.0%)
< 2 cm	58 (56.9%)	9 (28.1%)
≥ 2 cm	19 (18.6%)	15 (46.9%)
**Overall survival (days)**		
Range (median)	296-5630 (1098.5)	104-3750 (1165.5)
**Disease-free survival (days)**		
Range (median)	96-2884 (481.5)	97-2521 (369.5)
**Outcome**		
NED	13 (12.8%)	1 (3.1%)
AWD	3 (2.9%)	0 (0.0%)
DOD	86 (84.3%)	31 (96.9%)
**Sensitivity to treatment**		
Sensitive	67 (65.7%)	17 (53.1%)
Resistant	35 (34.3%)	15 (46.9%)
**Response to therapy**		
Complete remission	74 (72.6%)	22 (68.8%)
Other^a^	28 (27.4%)	10 (31.2%)

^a^) Other responses include: partial remission, progression, and no change.

NED – no evidence of disease; AWD – alive with disease; DOD – died of disease.

A response to chemotherapy was evaluated 3-4 weeks post chemotherapy, based on patient condition and CA125 levels. As to the assessment of clinical endpoints, complete remission (CR) was defined as a disappearance of all clinical and biochemical symptoms of ovarian cancer evaluated after completion of the first-line chemotherapy, and confirmed four weeks later [55]. Tumors were considered sensitive to treatment when disease-free survival of patients was longer than or equal to six months. Otherwise, tumors were presumed to be resistant [56]. Disease-free survival (DFS) time was assessed only for those patients who achieved complete remission. For the PC-treated subgroup, the follow-up time ranged from 104 to 3750 days (median: 1165.5 days); the respective values for the TP-treated subgroup ranged from 296 to 5630 days (median: 1098.5 days). All surviving patients had at least 2-year follow-up duration. Shorter follow-up times were due to earlier patient death. Completed observations were defined as those where the follow-up ended with patient death (OS) or relapse of the tumor (DFS).

This study was approved by the Bioethics Committee of Maria Sklodowska-Curie Institute - Oncology Center (ref. no. 39/2007).

### Cell cultures, plasmids, and transfection

Two ovarian cancer cell lines, A2780 (purchased from the European Collection of Cell Cultures, Porton Down, Salisbury, UK) and IGROV1 (kindly provided by dr J. Bernard, Institute G. Roussy, Villejuif, France), were cultured in RPMI-1640 medium (Thermo Fisher Scientific, Waltham, MA, USA) supplemented with 10% fetal bovine serum (FBS, Biochrom, Berlin, Germany) and 50 μg/ml gentamicin (Sigma-Aldrich, St. Louis, MO, USA) in a humidified incubator at 37°C with 5% CO_2_.

Initially, the *EMSY* gene silencing was performed with the use of the pGFP-B-RS vector (OriGene Technologies, Inc., Rockville, MD, USA) harboring one of three *EMSY*-specific shRNAs (Sh1, Sh2, Sh3) or a scrambled, non-silencing shRNA (SCR, a negative control), designed with the siRNA Wizard v3.1 web application (http://www.invivogen.com/sirnawizard/), see Supplementary Table 1 for details. In preliminary experiments (data not shown), we identified the Sh2 sequence as the strongest *EMSY* mRNA silencing molecule developed in our lab. We also proved that SCR shRNA does not diminish *EMSY* expression compared to the empty pGFP-B-RS vector. All shRNA-coding inserts were synthesized in the Institute of Biochemistry and Biophysics PAS (Warsaw, Poland) as two single-stranded, complementary DNA molecules. They were later annealed into double-stranded oligonucleotides containing BamHI and HindIII sticky ends, located upstream and downstream of the shRNA-coding region, respectively. Each of these oligos was ligated with the pGFP-B-RS vector earlier cleaved with the BamHI and HindIII restriction enzymes. The obtained constructs encoded the appropriate shRNA molecule and the GFP reporter protein. In addition, they harbored the kanamycin and blasticidin resistance genes allowing for a selection of transformants / transfectants in prokaryotic and eukaryotic cells, respectively. The GFP expression gave us the opportunity to discriminate transfected (green signal present) and non-transfected (no green signal) cells. All the constructs were sequenced twice using either the U6prom-F or SV40rev sequencing primer and the BigDye Terminator v3.1 Cycle Sequencing Kit (Life Technologies, Foster City, USA) supplemented with 5% DMSO and 40 μM dGTP.

1 × 10^6^ or 0.4 × 10^6^ cells per well (A2780 and IGROV1, respectively) were seeded in 6-well plate for 24h before transfection. Transfections were carried out using Lipofectamine-2000 (Invitrogen, Carlsbad, CA, USA) according to the manufacturer’s instructions. After transfection, cells were cultured for 48h with 8 nM paclitaxel (Sigma-Aldrich) or with 0.1% DMSO and numbers of the remaining cells were assessed in a Bürker chamber. Acquisition of fluorescence and non-fluorescence images of the cells, allowing for evaluation of transfection efficiency, was performed with the ZEISS LSM 800 confocal microscope with Airyscan, using a 10x objective (ZEISS, Oberkochen, Germany). Cell experiments were conducted in three independent biological replicates.

### DNA and RNA extraction

Fresh cancer specimens (obtained in the pathology laboratory) as well as the relevant blood samples anticoagulated with EDTA were snap-frozen in liquid nitrogen and stored at -70°C. Cryostat sections were cut, stained with hematoxylin and eosin, and then evaluated by the pathologist (JK) for a sufficient content of a tumor tissue (at least 85% tumor cell content). DNA from tumor and blood samples and both ovarian cancer cell lines was extracted with the use of QIAmp DNA Mini Kit (Qiagen, Hilden, Germany) according to the manufacturer’s instructions. RNA from clinical samples and cell lines was isolated using the RNeasy Plus Mini Kit (Qiagen), equipped with gDNA Eliminator columns. RNA quantity was measured with NanoDrop spectrophotometer (Thermo Fisher Scientific), and its quality was assessed on Agilent Bioanalyzer (Agilent Technologies, Santa Clara, CA, USA). RNA integrity numbers (RINs) of the samples ranged from 6.5 to 9.4.

### Molecular analysis of the *EMSY* gene

In clinical samples, all 20 protein-coding exons (from 2 to 21) of the *EMSY* gene were analyzed with the use of the polymerase chain reaction (PCR) followed by the single-strand conformational polymorphism (SSCP) and/or Sanger sequencing. Exons: 2, 4, 6, 10, 12, 13, 15, 16, 17 and 18 were initially screened for genetic alterations with SSCP and only the detected variants were sequenced. The remaining exons: 3, 5, 7, 8, 9, 9a (exon 9 was divided into two overlapping amplicons), 11, 14, 19, 20, 21 were analyzed by sequencing only. Some of the primers used were obtained from dr L. Hughes-Davies (Oncology Centre, Addenbrooke’s Hospital, Cambridge University Hospitals NHS Foundation Trust, Cambridge, UK). The remaining primers were designed in our laboratory using the Primer3 software (http://frodo.wi.mit.edu/cgi-bin/primer3/primer3_www.cgi). All primer sequences are listed in Supplementary Table 2. The *EMSY* reference genomic sequence was obtained from the NCBI Genome Browser, accession number: NC_000011.10. PCR mixtures were prepared according to the standard procedure (Applied Biosystems, Waltham, MA, USA). PCR reactions were carried out in a programmable thermal cycler (Eppendorf, Hamburg, Germany) with an initial denaturation step at 94°C for 10 min., followed by 36 cycles consisting of: denaturation (94°C, 30 sec.), annealing (55-62°C depending on an amplicon (Supplementary Table 2), 30 sec.), extension (72°C, 30-90 sec. depending on amplicon length). The final extension step was performed at 72°C for 7 min.

As mentioned before, some amplicons were initially screened with SSCP. Such PCR products were denatured with 0.1M NaOH (Sigma-Aldrich) containing 2 mM EDTA (Sigma-Aldrich) at 55°C for 15 min. After adding 95% formamide (Sigma-Aldrich), 0.05% xylene cyanol (Sigma-Aldrich) and 0.05% bromophenol blue (Sigma-Aldrich), the samples were immediately loaded on a polyacrylamide gel (1:39 *N,N’*-methylenebisacrylamide to acrylamide in 0.5 x TBE with 10% glycerol; Sigma-Aldrich). Electrophoresis was performed at 100 V for 16-24 hours at room temperature. DNA bands were visualized using a silver staining method compiled from several different procedures.

All samples, i.e., those screened and not screened with SSCP, were analyzed by Sanger sequencing. PCR products were purified with exonuclease I and alkaline phosphatase (Illustra ExoProStar, GE Healthcare Life Sciences, Little Chalfont, UK) for 18 min. at 37°C followed by 18 min. at 80°C to inactivate the enzymes. Then, the purified PCR products were sequenced with the use of BigDye Terminator v3.1 Cycle Sequencing Kit (Life Technologies) on ABI PRISM 3100 DNA sequencer (Life Technologies) according to the manufacturer’s recommendations.

Additionally, exons: 3, 5, 8, 9a, 11, 14, 20 of the *EMSY* gene were screened for genetic alterations in the A2780 and IGROV1 cells with the use of PCR followed by Sanger sequencing, as described above.

### Reverse transcription-quantitative PCR (RT-qPCR)-based studies of *EMSY* mRNA expression

All RT-qPCR reactions were carried out on the 7500 Fast Real-Time PCR System (Life Technologies) using three different house-keeping genes, *HGPRT*, *PPIA* and *GUSB*, as the reference for expression normalization. These genes were nominated from among 11 genes included on TaqMan Human Endogenous Control Plates (Life Technologies), based on their most stable expression in both the PC- and TP-treated subgroups. Expression of the reference genes was assessed for 8 randomly selected tumors from each subgroup. Next, the stability was calculated with the qBase^PLUS^ software (Biogazelle NV, Zwijnaarde, Belgium), utilizing an improved version of the geNorm algorithm [57, 58]. Gene expression was evaluated with the following TaqMan assays: id: Hs00220187_m1 (*EMSY*-specific, 6-FAM-labeled, Life Technologies), id: 4326321E (*HGPRT*-specific, VIC-labeled, Life Technologies), id: 4326316E (*PPIA*-specific, VIC-labeled, Life Technologies), and id: 4326320E (*GUSB*-specific, VIC-labeled, Life Technologies). RT-qPCR reactions were run in triplicates in a volume of 10 μl using TaqMan Universal Master Mix with uracil N-glycosylase (Life Technologies) and about 10-11 ng of total RNA, earlier reverse transcribed to cDNA with the High-Capacity cDNA Reverse Transcription Kit (Life Technologies). The obtained data were quantified using the delta delta Ct method for relative quantification of gene expression [59]. A tumor with the highest *EMSY* mRNA expression level and A2780/IGROV1 cells, transfected with the scrambled (SCR) shRNA and not treated with paclitaxel, were used as calibrators in experiments involving clinical samples and cell lines, respectively.

### Statistical analyses

The impact of *EMSY* gene polymorphisms and expression on the clinical outcome of ovarian cancer patients was assessed using the multivariate Cox proportional hazards model (prognostic value) or multivariate logistic regression model (predictive value). A presence of SNPs (categorical variables) and changes in the *EMSY* mRNA expression level (a continuous variable) were correlated with clinicopathological tumor characteristics, including: patient age (categorized by median split); residual tumor size; clinical stage (FIGO); histological grade (the last three parameters were categorized as shown in Table 5), and histological type (categorization: serous vs non-serous types). Statistical inference was conducted in the entire group of patients and also in subgroups with respect to the chemotherapy regimen applied. Additionally, all variables used in the multivariate Cox models were checked for proportionality of hazards (Supplementary Figure 5). In order to verify the discriminating capabilities of the Cox and logistic regression models, we performed their cross-validation in new data sets, generated from the original data by bootstrapping (with replacement) and subsequent comparison of areas under curves (AUCs) between the original and bootstrapped data sets, using the riskRegression package for R [60].

Afterwards, we used the Mann-Whitney U or Kruskal-Wallis tests to determine direct associations of *EMSY* gene expression (continuous data) with each variable included in the multivariate analyses and with SNP genotypes. In case of categorical data, i.e., SNPs, the same relationships were looked for but with the use of chi-square or Fisher’s exact probability tests, depending on a size of the analyzed groups.

Noteworthy, in the present study, *EMSY* mRNA expression was always treated as a continuous variable to avoid arbitrary categorization of data that could potentially lead to false statistical results. A tumor exhibiting the highest expression of the *EMSY* transcript was used as a calibrator. Thus, all the expression values ranged from 0 to 1. This approach allowed for approximate estimation of the risks based on the hazards ratios (HR) and odds ratios (OR) in a similar way as for categorical variables.

Time trends in overall survival, disease-free survival, complete remission and sensitivity to treatment were evaluated with the Mann-Kendall homogeneity test, and supported with the autocorrelation function (ACF) plots.

In all the tests, the initial statistical significance level (alpha) was set to 0.05. For the prognostic and predictive analyses, being carried out not only in the entire group of samples but also in the subgroups with different chemotherapy regimen used, the Bonferroni correction for multiple testing was applied, giving the new alpha value of 0.02 (0.05/3≈0.02).

Statistical analyses presented in this study were performed using either STATA, SAS or R software.

## SUPPLEMENTARY MATERIALS FIGURES AND TABLES


