# Supplementary material for: Clinical importance of the EMSY gene expression and polymorphisms in ovarian cancer
Source: Oncotarget. 2018 Apr 3;9(25):17735–55. doi: 10.18632/oncotarget.24878 (PMC5915152; doi:10.18632/oncotarget.24878)
Supplement: Supplementary file 1 [file oncotarget-09-17735-s001.pdf]

Clinical importance of the EMSY gene expression and polymorphisms in ovarian cancer

SUPPLEMENTARY MATERIALS

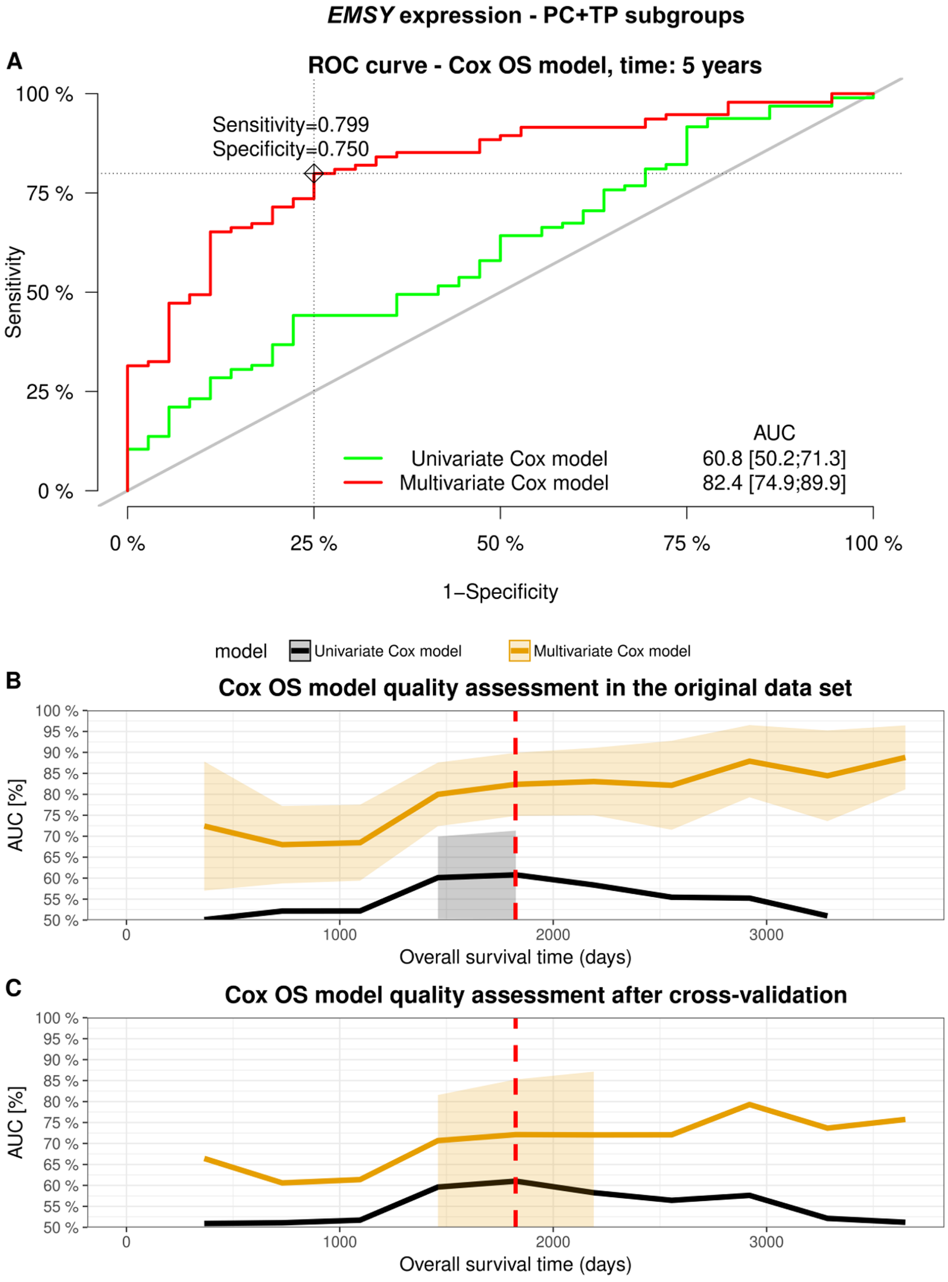

Supplementary Figure 1: Performance comparison of the Cox regression models allowing for assessment of the risk of death in ovarian cancer patients treated with PC or TP, depending on either a single independent variable (*EMSY* mRNA expression (exp)) – the univariate model or six independent variables (exp, residual tumor size, patient age, and tumor: FIGO stage, grade, histological type) – the multivariate model. **(A)** shows a time-dependent ROC curve for each model, with an optimal cutpoint based on the Youden index. Sensitivity and specificity for this cutpoint are also provided. In addition, area under the ROC curve (AUC) values [%] with 95 % CI are listed. **(B)** depicts how the AUC value changes in time, and a red dashed line marks the same time point which was used in the time-dependent ROC curve. **(C)** shows how discriminating abilities of each model change after cross-validation involving 100-fold bootstrapping (with replacement) of the original data set. The bigger the AUC, the higher the performance of a model.

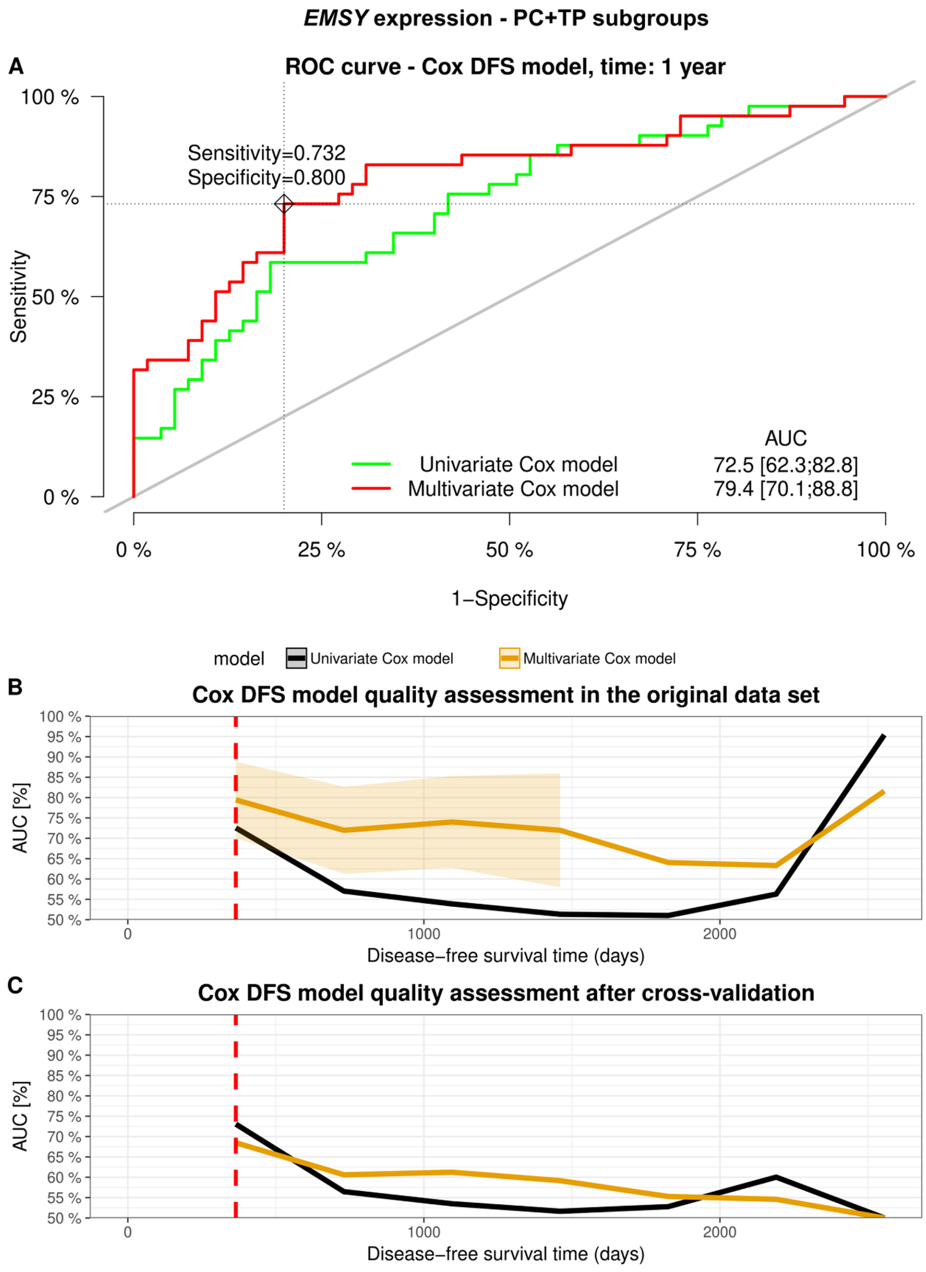

Supplementary Figure 2: Performance comparison of the Cox regression models allowing for assessment of the risk of relapse in ovarian cancer patients treated with PC or TP, depending on either a single independent variable (*EMSY* mRNA expression (exp)) – the univariate model or six independent variables (exp, residual tumor size, patient age, and tumor: FIGO stage, grade, histological type) – the multivariate model. (A) shows a time-dependent ROC curve for each model, with an optimal cutpoint based on the Youden index. Sensitivity and specificity for this cutpoint are also provided. In addition, area under the ROC curve (AUC) values [%] with 95 % CI are listed. **(B)** depicts how the AUC value changes in time, and a red dashed line marks the same time point which was used in the time-dependent ROC curve. **(C)** shows how discriminating abilities of each model change after cross-validation involving 100-fold bootstrapping (with replacement) of the original data set. The bigger the AUC, the higher the performance of a model.

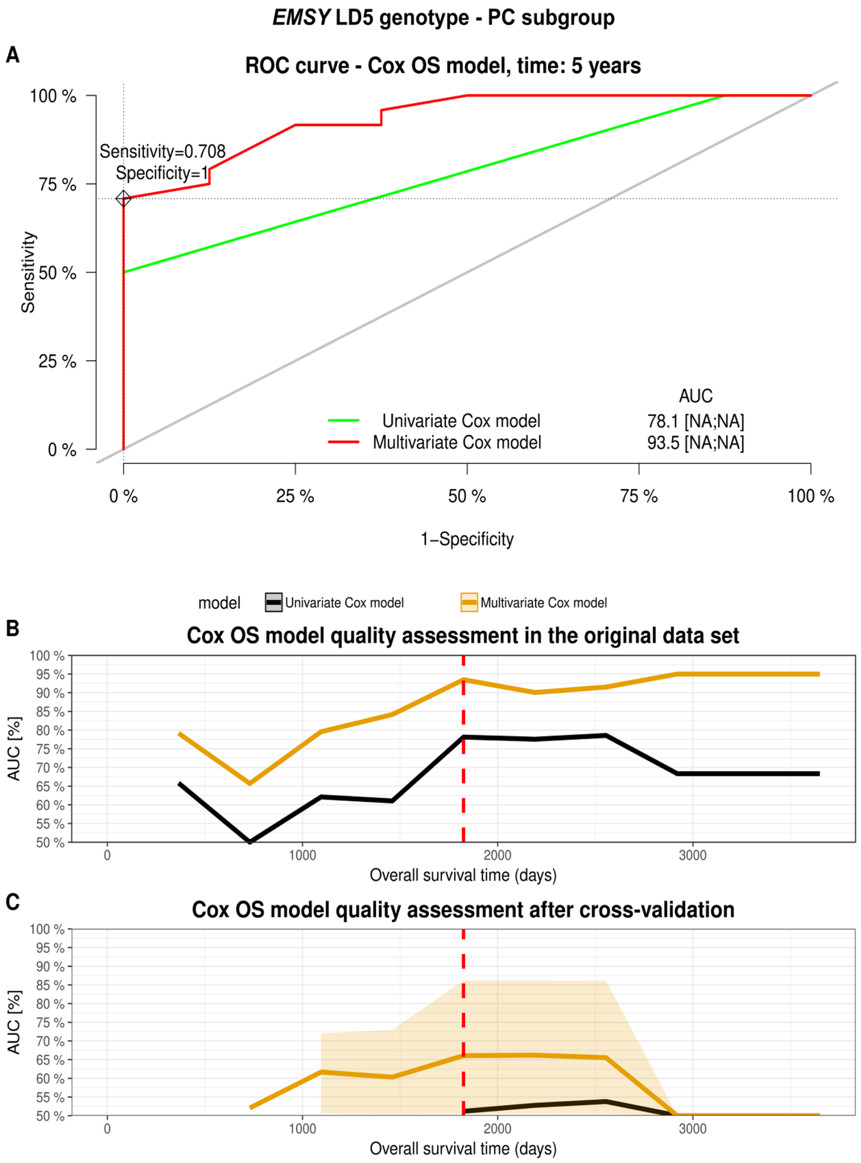

Supplementary Figure 3: Performance comparison of the Cox regression models allowing for assessment of the risk of death in ovarian cancer patients treated with PC, depending on either a single independent variable (*EMSY* LD5 genotype (LD5)) – the univariate model or six independent variables (LD5, residual tumor size, patient age, and tumor: FIGO stage, grade, histological type) – the multivariate model. (A) shows a time-dependent ROC curve for each model, with an optimal cutpoint based on the Youden index. Sensitivity and specificity for this cutpoint are also provided. In addition, area under the ROC curve (AUC) values [%] with 95 % CI are listed. **(B)** depicts how the AUC value changes in time, and a red dashed line marks the same time point which was used in the time-dependent ROC curve. **(C)** shows how discriminating abilities of each model change after cross-validation involving 3-fold bootstrapping (with replacement) of the original data set. More bootstrapping rounds were not performed due to a relatively small number of cases in the data set. The bigger the AUC, the higher the performance of a model.

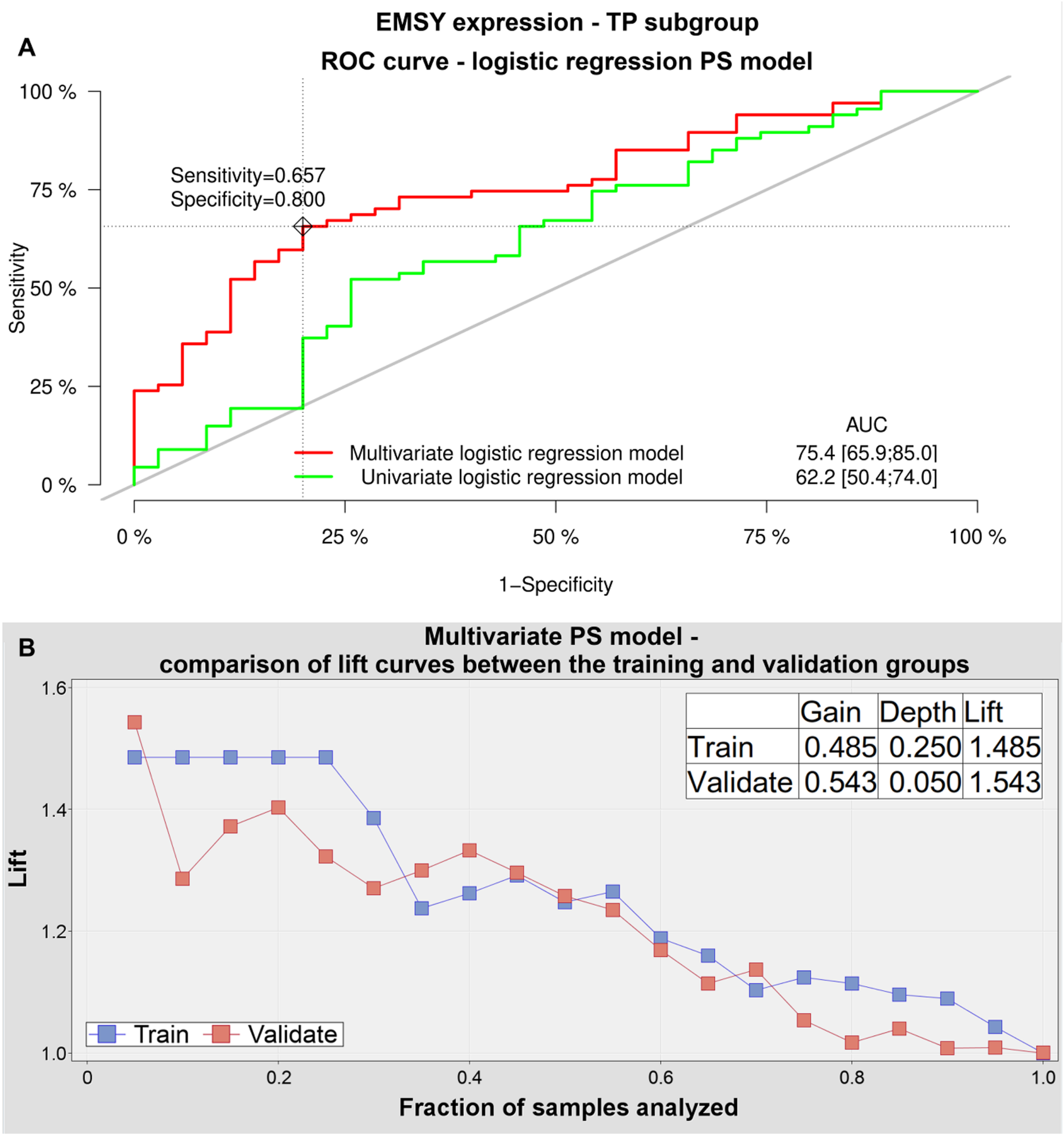

Supplementary Figure 4: Performance comparison of the logistic regression models allowing for assessment of the chance for sensitivity to chemotherapy in ovarian cancer patients treated with TP, depending on either a single independent variable (*EMSY* mRNA expression (exp)) – the univariate model or six independent variables (exp, residual tumor size, patient age, and tumor: FIGO stage, grade, histological type) – the multivariate model. (A) shows a ROC curve for each model, with an optimal cutpoint based on the Youden index. Sensitivity and specificity for this cutpoint are also provided. In addition, area under the ROC curve (AUC) values [%] with 95 % CI are listed. It was impossible to plot the AUC curves for these models, since they are time-independent. Nevertheless, the AUC values after cross-validation with 10-fold bootstrapping equaled 67.5 % and 73.2 % for the univariate and multivariate model, respectively, and were similar to those shown in (A). **(B)** depicts lift curves for the same multivariate model. The original data set was randomly divided into two subgroups of the same size. One of these subgroups was then used as a training and the other as a validation data set. Overlapping of the lift curves suggests that performance of the model is comparable for both data sets.

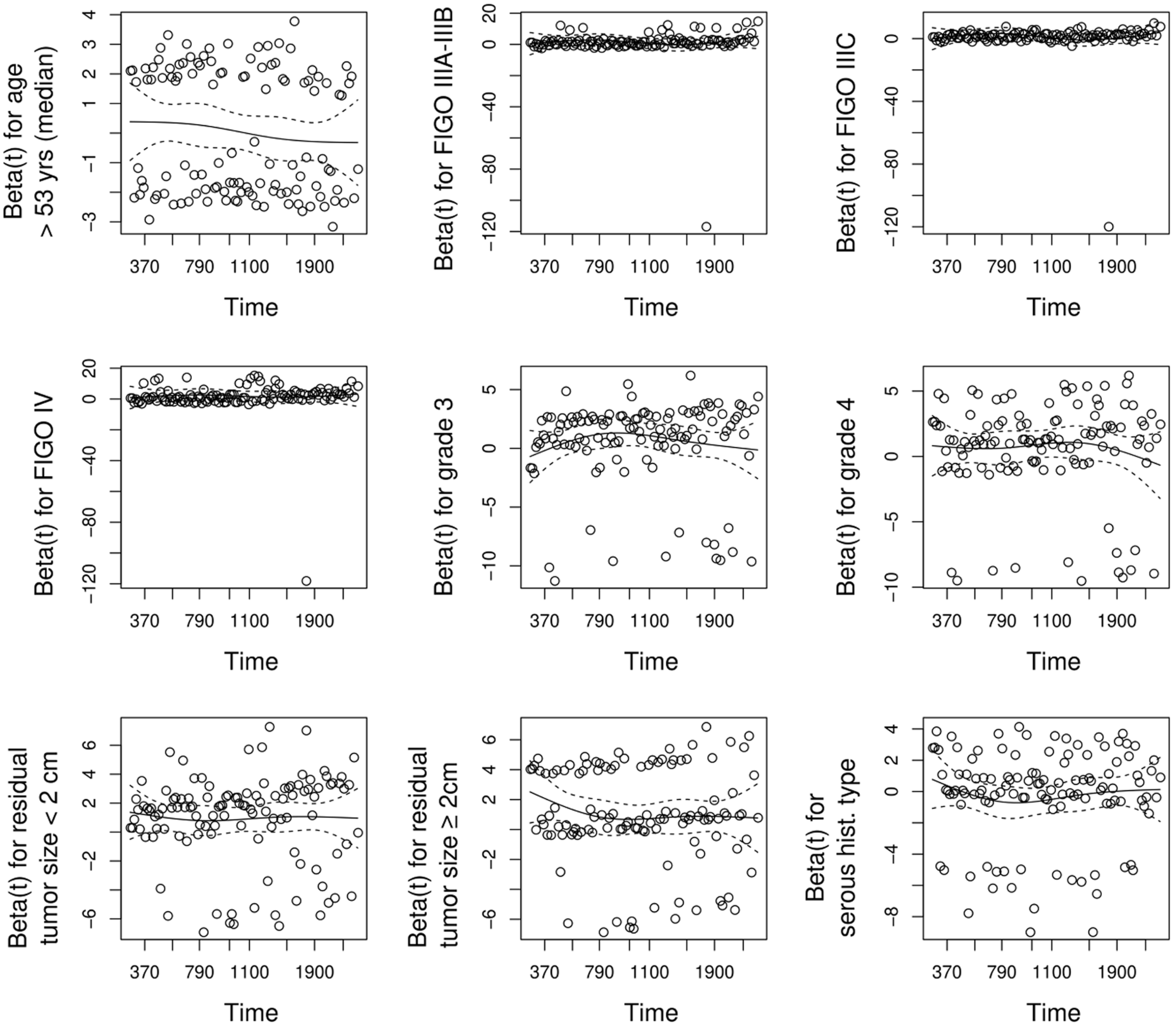

Supplementary Figure 5: Assessing the proportionality of hazards (death) for independent variables used in multivariate Cox regression models. All hazards were proportional (p-values > 0.05). This plot is a graphical representation of these results. Hazards of relapse were also proportional for all the variables (data not shown).

Supplementary Table 1: Sequences of oligonucleotides used for generation of inserts encoding *EMSY*-silencing and control (scrambled) shRNAs

Primer name	Sequence [5′ → 3′]
Sh1 sense strand[a]	GATCG**GACCAAGTTACAGTATGTCTGGACCTAAT**TCAAGAGATTAGGTCCAGACATACTGTAACTTGGTCTTTTTTGA
Sh1 complementary strand[a]	AGCTTCAAAAAA**GACCAAGTTACAGTATGTCTGGACCTAAT**CTCTTGAATTAGGTCCAGACATACTGTAACTTGGTCC
Sh2 sense strand[a]	GATCG**GAGCAGTAAACGATGAACGGTTAACAACA**TCAAGAGTGTTGTTAACCGTTCATCGTTTACTGCTCTTTTTTGA
Sh2 complementary strand[a]	AGCTTCAAAAAA**GAGCAGTAAACGATGAACGGTTAACAACA**CTCTTGATGTTGTTAACCGTTCATCGTTTACTGCTCC
Sh3 sense strand[a]	GATCG**GGTAGCAGAGGCTGGTAATTCATCTAT**TCAAGAGATAGATGAATTACCAGCCTCTGCTACCTTTTTTGA
Sh3 complementary strand[a]	AGCTTCAAAAAA**GGTAGCAGAGGCTGGTAATTCATCTAT**CTCTTGAATAGATGAATTACCAGCCTCTGCTACCC
Sh SCR (scrambled, negative control)sense strand[a]	GATCG**GGAGCAATATCGTGGATGAAACGGTGAAA**TCAAGAGTTTCACCGTTTCATCCACGATATTGCTCCTTTTTTGA
Sh SCR (scrambled, negative control)complementary strand[a]	AGCTTCAAAAAA**GGAGCAATATCGTGGATGAAACGGTGAAA**CTCTTGATTTCACCGTTTCATCCACGATATTGCTCCC

[a]) shRNA-coding sequences: sense regions were highlighted in bold type, whereas antisense regions were underlined; sticky ends, specific to BamHI/HindIII digestion, were double-underlined.

Supplementary Table 2: Sequences of PCR primers used for amplification of the *EMSY* gene

Exon	Sequence of the forward primer	Sequence of the reverse primer	Annealing temperature
2	TGGAAAGTGTGGTGGTGAGA	TGGGAACTGTATCTCCAAAGAA	56°C
3	TTTTGCCCTGAAGTTTCACTA	AAAATCCCCCAAAATGAAAT	58°C
4	AGCAATCTTGTCCCCCTTCT	AAGCTTGCCTAACCAAATGC	55°C
5	CCTAAGTGTGTGACATTCCT	GTCATCCATTAAGGCCTTCT	56°C
6	TATGCCTGGACAGTTTTGGT	CAAGAAGGCCCTGATAACAC	58°C
7	TGGCTTAAGACAGAGAGAGGACA	TGTGAACATCCCCAGCATAA	56°C
8	GGCACATAGTAGGTGCTCAA	CGACCATCTTGACATTCTCA	56°C
9	CTGACACTTACTAGGTGCTCA	TGAAGAGACATACCGTGTTCAT	55°C
9a	CCATCCAAATCAAACAGGAG	GACAAATTGAATCCAGCAATG	58°C
10	TGGGTTGGTATAAGGGACAT	TGAATAAATGAACTGATGCTCAA	58°C
11	ATGTGTTGAAAACTGGCTGTAA	AAAAGCAACTAAAGGTTCTTTCCA	55°C
12	TCCTTAACTCAGGCCTCCTT	GCTGGTCTTTAAATCCTCTGTG	58°C
13	TTTTTCAGTGAGAGGACTTTATCA	AAGAGCCAAGAAAAATCATCA	58°C
14	TTGCACTCTGGCATGTACCA	TCAGCTCACCGATTTAACATCA	56°C
15	GAAACTTTGTTTATAGGGTTTGTTTT	TTTTAGCACAACCCGTCTCT	55°C
16	GTGGACAACTCTGGTTTTGG	CACACAATCATCCCATGAAA	58°C
17	TCAAGCAGAGGCCAGTATTC	TAGAGCGCATCCATTTAAGC	58°C
18	GGGCAGACTGTGGTCTTCTG	ACGTTCCAATTATTTCTCCTCTT	56°C
19	CGAAGCACTTACGTCTGAGA	GCACTGTTGGTCAATGCTGA	55°C
20	GGCTGGCTGAACAAAGGTTC	TGTGCTGGGTGACAATGTC	56°C
21	TTCTCCTGTGTCTTCTCTTCCA	AAGGGTTTCCCTGGACACTT	62°C
